# Atypical Teratoid/Rhabdoid Tumor of the Lateral Ventricle: A Case Series and Experience with Molecular Subtyping-Guided Immunotherapy

**DOI:** 10.3390/neurolint18040074

**Published:** 2026-04-21

**Authors:** Haohan Wang, Zesheng Ying, Zhuo Zhi, Nijia Zhang, Jia Wang, Nan Zhang, Yingjie Cai, Ming Ge

**Affiliations:** 1 Department of Neurosurgery, Beijing Children’s Hospital, Capital Medical University, National Center for Children’s Health, Beijing 100045, China; 13463096262@163.com (H.W.); yingzesheng2022@163.com (Z.Y.); 13519745647@163.com (Z.Z.); zoro1992612@163.com (N.Z.); ojiageo@sina.com (J.W.); 2 Department of Pathology, Beijing Children’s Hospital, Capital Medical University, National Center for Children’s Health, Beijing 100045, China; nanwnan@163.com

**Keywords:** lateral ventricle, atypical teratoid rhabdoid tumor, epigenomics, immunotherapy, case report

## Abstract

Background: Atypical teratoid/rhabdoid tumors (AT/RT) are rare, highly aggressive pediatric central nervous system (CNS) malignancies. AT/RT of the lateral ventricle is an exceptionally rare subgroup, with only 11 reported cases. *SMARCB1* inactivation is the primary molecular feature of AT/RT. Current consensus is to classify AT/RT based on methylation and molecular profiles into the following subgroups: AT/RT-*TYR*, AT/RT-*SHH*, AT/RT-*MYC*, and a potentially distinct *SMARCA4*-deficient subtype. AT/RT-*MYC* exhibits high levels of CD8^+^ tumor-infiltrating lymphocytes, indicating immunogenic potential. Case presentation: We report three pediatric cases presenting with intracranial hypertension and seizures. Diagnosis was confirmed via histopathology and molecular profiling. Interventions included gross total resection, chemotherapy, radiotherapy, and combined immune checkpoint inhibitors (pembrolizumab and ipilimumab). Outcomes varied from rapid progression to 3-year recurrence-free survival. A cohort of 14 pediatric patients with lateral ventricle AT/RT, comprising 3 institutional cases and 11 cases identified from the PubMed database, was evaluated through a narrative synthesis. Conclusions: These advancements highlight the crucial role of molecular subtyping in tailoring personalized treatments, including epigenetic modifiers and immune-based regimens. However, clinical validation is essential to establish standardized protocols. Integrating genomic, epigenetic, and immune microenvironment profiling may enhance risk assessment and treatment precision, ultimately improving survival and quality of life in pediatric patients.

## 1. Introduction

Atypical teratoid/rhabdoid tumor (AT/RT) are rare, highly aggressive pediatric central nervous system (CNS) malignancies primarily defined by *SMARCB1* (*INI1*) inactivation. These tumors account for approximately 1% to 2% of all pediatric CNS malignancies [1]. Recent molecular profiling has classified AT/RT into distinct subgroups (AT/RT-*TYR*, -*SHH*, and -*MYC*) [2]. The AT/RT-*MYC* subgroup is notably associated with a heightened immunogenic potential, driving therapeutic interest in novel immune-based regimens, such as immune checkpoint inhibitors.

Despite these molecular advancements, the clinical management of AT/RT remains exceptionally challenging, with a poor overall prognosis under conventional surgery, radiotherapy, and chemotherapy. AT/RT originating in the lateral ventricle constitute an exceedingly rare anatomical subgroup, yet they present a unique clinical opportunity. Emerging evidence suggests that the anatomical site of AT/RT may be intimately linked to its cellular lineage of origin within the subventricular zone (SVZ), potentially dictating both molecular subtype and therapeutic vulnerability. Currently, there is a critical lack of data regarding the unique biological behaviors or prognostic associations of ventricular AT/RT. The aim of this study is to summarize the clinicopathological and molecular characteristics of lateral ventricle AT/RT through a retrospective case series (*n* = 3) and systematic literature review (*n* = 11). Our findings underscore the critical role of molecular subtyping in guiding personalized treatment, particularly the application of novel immune-based strategies in this highly aggressive tumor.

## 2. Materials and Methods

### 2.1. Retrospective Case Series

A retrospective analysis of patients aged <18 years who underwent tumor resection at the Department of Neurosurgery, Beijing Children’s Hospital, between January 2016 and 2024, was conducted. The Pathology Department of Xuanwu Hospital, Capital Medical University, confirmed the postoperative histopathological diagnosis of atypical teratoid/rhabdoid tumor (AT/RT). Subsequent treatment details of each patient were systematically recorded through a combination of regular outpatient clinic visits with follow-up MRI (for surviving cases) and supplementary long-term telephone follow-ups. Outcome measures included Overall Survival (OS, time from surgery to death or last contact) and Progression-Free Survival (PFS, time to first progression or death). Disease status was monitored via outpatient MRI and supplementary telephone interviews. Data on demographics, clinical presentation, neuroimaging, histopathology, treatment, and outcomes were collected. This study was approved by the Ethics Committee of Beijing Children’s Hospital.

Somatic copy number variations (SCNVs) were analyzed using data from a targeted next-generation sequencing (NGS) panel assay encompassing 1295 genes associated with brain tumors (Genetron Health, Beijing, China). Copy number (CN) analysis was performed (Genetron Health, Beijing, China) by comparing the tumor sample’s sequencing depth with a reference control; subsequently, the results were verified to determine *SMARCB1* deletion.

### 2.2. Literature Review Strategy

Following this retrospective case series, a PubMed/MEDLINE search was conducted for English-language articles published between 2004 and 2025 using the keywords: (“lateral ventricle” OR “ventricular”) AND (“atypical teratoid/rhabdoid tumor” OR “AT/RT”). The terms were combined using the Boolean operators ‘AND’ and ‘OR’ to ensure a comprehensive retrieval of relevant cases. The inclusion criteria were: (1) histologically confirmed AT/RT; (2) primary tumor location in the lateral ventricle; and (3) patients aged 0–18 years. Literature was excluded based on the following pre-defined criteria: (1) duplicated cases; (2) non-peer-reviewed sources, including conference abstracts and unpublished reports; and (3) cases where the primary tumor site could not be definitively localized to the lateral ventricle. Retrieved clinical studies were systematically screened according to the predefined inclusion and exclusion criteria, after which a narrative synthesis was performed on the extracted data.

## 3. Result

### 3.1. Case Presentation

#### 3.1.1. Case 1

**Figure 1:**
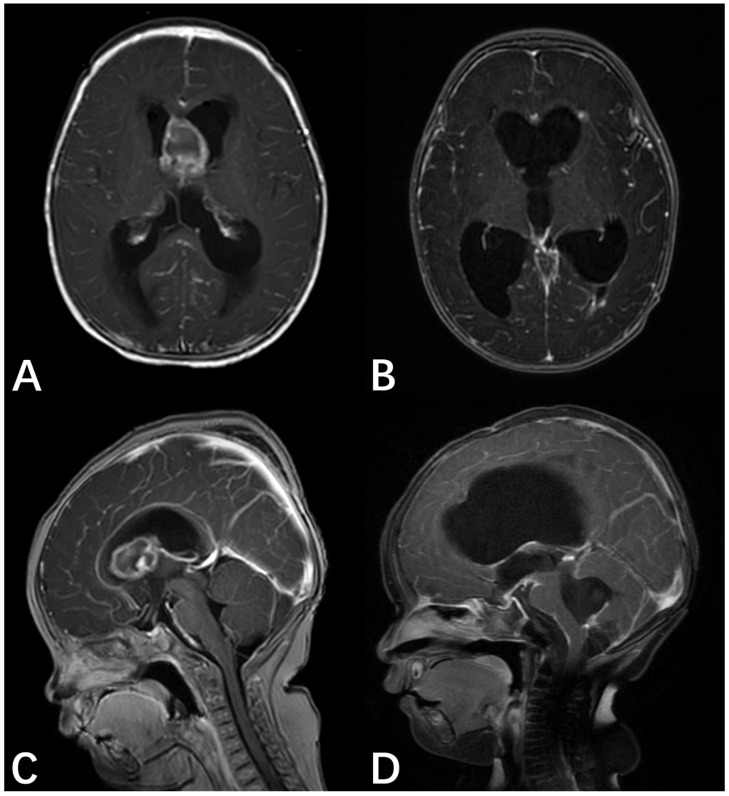
MRI for case 1. (**A**) Preoperative axial T1-weighted image with gadolinium enhancement. (**B**) Postoperative axial T1-weighted image with gadolinium enhancement. (**C**) Preoperative sagittal T1-weighted image with gadolinium enhancement, revealing a 1.8 × 2.4 × 1.8 cm mass located between the bilateral frontal horns of the lateral ventricles, characterized by heterogeneous signal intensity. (**D**) Sagittal T1-weighted image with gadolinium enhancement obtained at 8-month follow-up, showing no evidence of tumor dissemination or residual mass enlargement.

**Figure 2:**
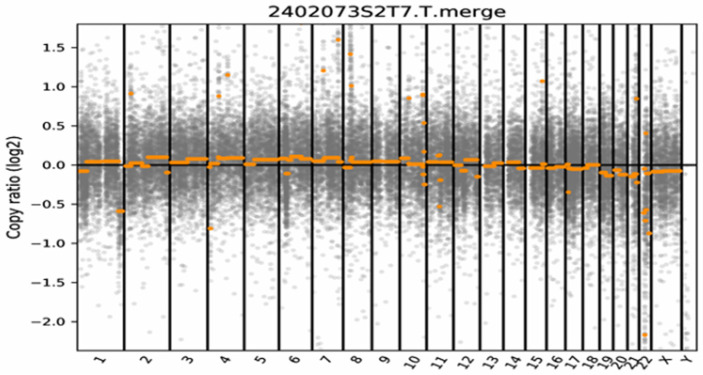
Results of somatic copy number variation (SCNV) analysis. The x-axis displays the chromosomes (autosomes 1–22 and sex chromosomes X, Y), separated by vertical black lines. The orange dots and horizontal lines represent the log2 copy ratio of the sequenced genomic segments. The log2 copy ratio remained near zero across the majority of the genome, indicating an absence of large-scale chromosomal aneuploidy or significant segmental gains/losses. Notably, a focal deletion at the 22q11.2 locus was identified, corresponding to the biallelic inactivation of the *SMARCB1* gene. This genomic stability, coupled with isolated *SMARCB1* loss, is consistent with the classic molecular signature of atypical teratoid/rhabdoid tumors.

A 6-month-old female infant presented with severe vomiting, irritability, and seizures worsening over the course of one week. The patient underwent emergency bilateral external ventricular drainage (EVD) at a local facility and was subsequently transferred to our institution for definitive tumor management. An emergency CT scan revealed an intracranial mass causing hydrocephalus; an MRI showed an oval-shaped mass with variable signal intensity located between the anterior horns of the lateral ventricles, resulting in ventricular dilation and periventricular edema. Gadolinium-enhanced T1-weighted imaging (T1WI) revealed a significant peripheral rim enhancement of the mass (Figure 1A,C). The patient had a significant family history of malignancy. Specifically, the father was diagnosed with high-grade esophageal intraepithelial neoplasia, the paternal grandmother had endometrial cancer, and the great-grandmother was diagnosed with esophageal cancer. On the fifth hospital day, the patient underwent an ultrasound-guided right frontal craniotomy for complete resection of the lesion. Postoperative spinal MRI revealed metastatic lesions in the brainstem and spinal regions. Histopathological examination confirmed a lateral ventricle AT/RT. Probe hybridization and high-throughput sequencing showed widespread deletions of the *SMARCB1* gene with a copy number (CN) of 0.44 and deletions of the *NF2* gene, TMB score = 0 Muts/Mb, MSI score = 21.55%. Germline mutation testing showed a deletion at nucleotides 604 and 605 in the *LZTR1* gene. The molecular profile featured a biallelic *SMARCB1* loss, a defining *NF2* deletion, and a predisposing *LZTR1* germline mutation (Figure 2).

This patient underwent postoperative adjuvant chemotherapy, including 2 cycles of the ifosfamide, carboplatin, etoposide (ICE) regimen and 1 cycle of vincristine, actinomycin D, and cyclophosphamide, combined with intrathecal topotecan. However, a follow-up spinal MRI performed 20 days after discharge from the final chemotherapy cycle revealed that the spinal dissemination had not improved. Given the aggressive nature of the disease and the inadequate response to chemotherapy, after thorough communication with the parents, whole ventricular radiotherapy was administered (total of 20 fractions, 1.6 Gy per fraction). Two months later, a follow-up spinal MRI review showed no significant improvement and a tendency for the lesions to increase. Given the early failure of conventional adjuvant therapy and the aggressive nature of the disease, immunotherapy was initiated based on the patient’s molecular profiling results, including (i) pembrolizumab at 2 mg/kg for 7 cycles, approximately every 3 weeks, (ii) bevacizumab at 10 mg/kg for 4 cycles, approximately every 2 weeks, and (iii) ipilimumab at 1 mg/kg for 2 cycles, every 3 weeks. At the 8-month follow-up, the patient showed no improvement in hydrocephalus; rather, the condition worsened gradually, leading to an endoscopic third ventriculostomy at an external hospital. Brain MRI revealed no evidence of residual tumor or recurrence at the primary site compared with the post-radiotherapy imaging. Furthermore, the previously documented spinal metastases remained stable in size, indicating that the disease was well-controlled under the current multimodal salvage therapy (Figure 1B,D).

**Figure 3:**
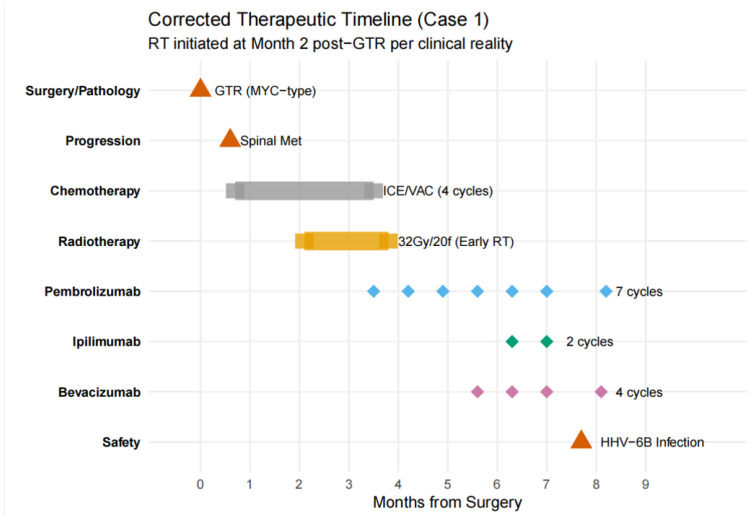
Comprehensive therapeutic timeline of Case 1. This plot illustrates the clinical narrative and treatment sequence over a 9-month period. Key milestones include initial GTR, early spinal metastasis, chemotherapy, radiotherapy, and a novel immunotherapy regimen (Pembrolizumab and Ipilimumab) combined with Bevacizumab. The timeline also captures the occurrence of a HHV-6B infection, providing a transparent view of both therapeutic efficacy and clinical safety.

To provide a clear overview of the complex clinical course of Case 1, a comprehensive therapeutic timeline was constructed (Figure 3). As illustrated, following initial surgery and rapid spinal progression, the patient underwent a multimodal salvage strategy.

#### 3.1.2. Case 2

**Figure 4:**
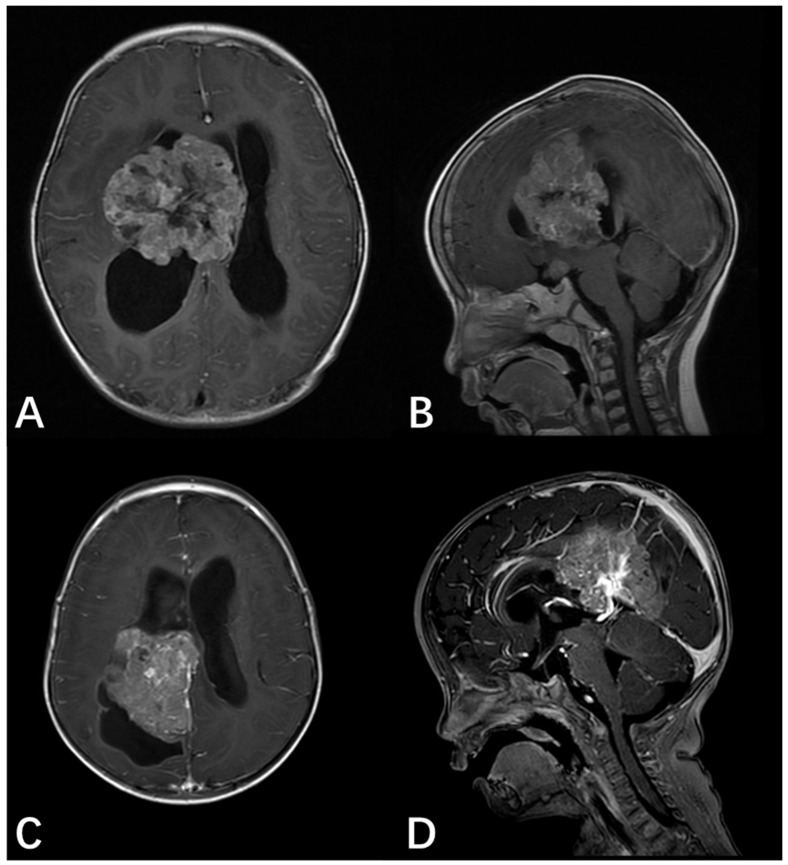
Preoperative MRI for Case 2 and Case 3. (**A**) Preoperative axial T1-weighted image with gadolinium enhancement for Case 2. (**B**) Preoperative sagittal T1-weighted image with gadolinium enhancement for Case 2, revealing a large, irregular, and nodular mass occupying the right lateral ventricle and extending toward the basal ganglia. (**C**) Preoperative axial T1-weighted image with gadolinium enhancement for Case 3. (**D**) Preoperative sagittal T1-weighted image with gadolinium enhancement for Case 3, revealing a large (approximately 6.5 × 6.0 × 5.5 cm) cystic-solid mass in the right lateral ventricle body, extending into the frontal and parietal lobes. The solid components exhibited intense heterogeneous enhancement.

**Figure 5:**
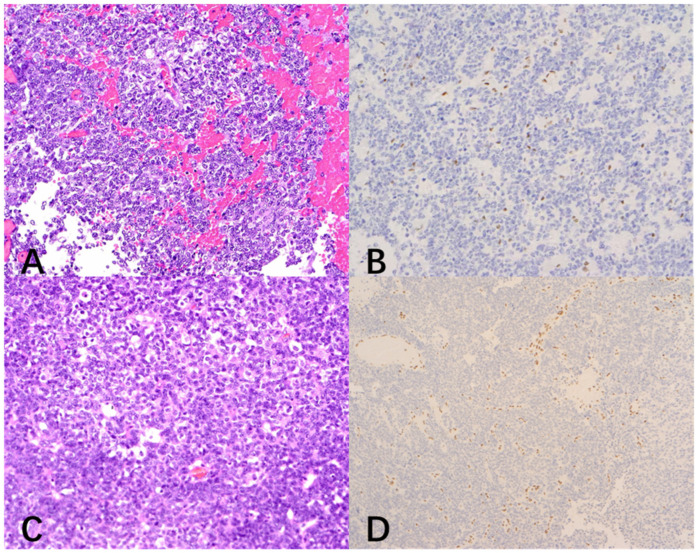
Histopathological findings and immunohistochemical analysis for INI1/*SMARCB1* (magnification ×200). (**A**) Hematoxylin and Eosin (H&E) staining of the resected tumor for Case 2. (**B**) Immunohistochemical analysis for INI1/*SMARCB1* in Case 2. (**C**) Hematoxylin and Eosin (H&E) staining of the resected tumor for Case 3. (**D**) Immunohistochemical analysis for INI1/*SMARCB1* in Case 3. Tumor cells show a complete loss of nuclear expression (negative staining, light blue/purple) for INI1.

A 2-year-4-month-old male presented with a 3-week history of progressive gait ataxia and left upper extremity monoparesis, culminating in a 3-day history of severe headache and vomiting, indicative of raised intracranial pressure. Gadolinium-enhanced MRI revealed a large, irregular, nodular, “mulberry-like” mass occupying the right lateral ventricle and extending toward the basal ganglia, resulting in severe obstructive hydrocephalus (Figure 4A,B). Preoperative spinal MRI excluded leptomeningeal dissemination. On the third day of hospitalization, the patient rapidly deteriorated with generalized tonic seizures, opisthotonus, and anisocoria (right pupil 4 mm, left pupil 2.5 mm), suggestive of descending transtentorial herniation. An emergency EVD was immediately performed to restore pupillary symmetry. The patient subsequently underwent a minimally invasive right frontoparietal craniotomy, achieving complete microscopic resection of the intraventricular tumor. Postoperative histopathology confirmed lateral ventricle AT/RT, characterized by loss of INI1 expression and a high Ki-67 proliferation index (~60%) (Figure 5A,B). The postoperative course was complicated by refractory status epilepticus and intracranial infection (meningitis), requiring aggressive Pediatric Intensive Care Unit (PICU) management, mechanical ventilation, and both systemic and intrathecal antibiotic therapy. The patient commenced conventional adjuvant chemotherapy and radiotherapy post-stabilization. However, at the 3-month follow-up, the patient succumbed to the disease due to tumor progression.

#### 3.1.3. Case 3

An 11-month-old male pediatric patient was admitted following the incidental discovery of a massive intracranial lesion after a minor head trauma accompanied by vomiting. Preoperative MRI revealed a large (approximately 6.5 cm × 6.0 cm × 5.5 cm) cystic-solid mass in the right lateral ventricle body, extending into the frontal and parietal lobes. The solid components exhibited intense heterogeneous enhancement. The tumor caused a significant mass effect, compressing the thalamus and corpus callosum (Figure 4C,D). Preoperative spinal MRI excluded leptomeningeal dissemination. The patient underwent microscopic gross total resection (GTR) of the deep subventricular tumor. Intraoperative findings confirmed that the tumor originated from the lateral ventricular wall. Postoperative histopathology confirmed a lateral ventricle AT/RT, characterized by the presence of rhabdoid cells and a distinct immunophenotypic loss of INI1 (Figure 5C,D). The postoperative course included PICU management for transient respiratory support and correction of hematological issues. Following recovery from surgery, the patient returned to a local hospital where they received postoperative adjuvant chemotherapy and stem cell transplantation (SCT). At the 3-year long-term follow-up, the patient maintained a favorable functional status with no signs of tumor recurrence or residual disease.

### 3.2. Pooled Analysis of Institutional and Literature Cohorts

A total of 14 lateral ventricle AT/RT cases, including the three new cases, are summarized in Table 1. The median age at diagnosis for the entire cohort (*n* = 14) was 42 months (range, 2 months–15 years). Clinical follow-up data were available for 11 patients (78.6%), with a median follow-up duration of 21 months (range, 2 to 48 months). At the latest follow-up, 5 patients (35.7%) survived, while 7 patients (50.0%) had died. Due to the limited sample size and missing follow-up data for three cases (two with unknown outcomes and one with unknown survival duration), a formal Kaplan–Meier median overall survival could not be definitively calculated. GTR was accomplished in 71.4% (10/14) of cases, in conjunction with chemotherapy in 71.4% (10/14) and radiotherapy in 50.0% (7/14). In comparison with posterior fossa AT/RT, lateral ventricular cases showed a relatively favorable short-term outcome. However, given the small sample size and missing survival data for some cases, this should be interpreted as a preliminary clinical observation rather than a definitive survival benefit, which requires further validation in larger, multi-center studies.

## 4. Discussion

Primary AT/RT originating in the lateral ventricle is exceptionally rare, with fewer than 14 cases reported in the global literature to date. This malignancy predominantly affects children under 3 years of age, with a slight male predominance (M:F = 9:5) [14,15,16,17]. This study presents the largest single-center case series of lateral ventricle AT/RT. It notably provides the first detailed report of a patient who underwent comprehensive tumor genetic testing followed by combination immune checkpoint inhibitor therapy. This work holds significant clinical value by offering support for innovative immunotherapy and targeted treatment strategies for this challenging tumor.

AT/RT present variably without specific symptoms; manifestations correlate with tumor location. In the posterior cranial fossa, AT/RT may exhibit cerebellar dysfunction, including ataxia and gait disturbance, while cranial nerve involvement can lead to corresponding symptoms [18,19]. Lateral ventricle AT/RT commonly presents with intracranial hypertension-related symptoms, followed by seizures and muscle weakness. The disease typically progresses rapidly, with the interval from symptom onset to diagnosis ranging from 1 week to 4 months. Children with posterior fossa AT/RT tend to experience more severe hydrocephalus symptoms due to the smaller space in the fourth ventricle compared to the lateral ventricle [19,20]. The clinical presentation and progression of AT/RT highlight its aggressive nature and the associated neurological complications.

Given that AT/RT are extremely rare in the lateral ventricle, they are not often included in the initial differential diagnosis of intraventricular masses. Nevertheless, clinicians’ suspicion for AT/RT should increase with the observation of specific imaging hallmarks. On CT, AT/RT often present as heterogeneous hyperdense masses, with calcification, hemorrhage, and necrosis. On MRI, these lesions typically appear isointense or hypointense on T1WI, demonstrate restricted diffusion with hyperintensity on diffusion-weighted imaging (DWI), and show low apparent diffusion coefficient (ADC) values, indicating high tumor cellularity [7,21,22]. Peritumoral edema and significant mass effect may also be observed, reflecting the infiltrative growth pattern of the tumor [23].

Choroid plexus tumors (CPTs) may also exhibit invasive features; however, AT/RT’s tendency to disseminate leptomeningeally and the typically younger age at diagnosis can help distinguish them [24,25]. Meningiomas, which may also appear as enhancing intraventricular masses on MRI, can be differentiated from AT/RT by the presence of the dural tail sign in up to 72% of cases and a higher average age at presentation [26,27]. Although imaging studies play a pivotal role in early diagnosis, definitive diagnosis of AT/RT relies on postoperative histopathological and molecular analyses.

Due to the rarity and aggressive nature of AT/RT, standardized international clinical practice guidelines remain limited, resulting in heterogeneous treatment protocols across institutions. Nevertheless, a consensus emphasizes a comprehensive multimodal strategy, which is fundamentally underpinned by early maximum surgical resection, specifically GTR. GTR is widely recognized as a crucial independent prognostic factor in AT/RT patients [27,28]. Furthermore, studies suggest that the anatomical tumor location may influence AT/RT prognosis [27,28]. Consistent with published literature, GTR was achieved in 71.4% of the cases in the lateral ventricle AT/RT series of this report. The generally dismal prognosis of AT/RT is reflected in a pooled 2-year OS rate of approximately 37.1%, as reported in systematic reviews [27]. The systematic review of lateral ventricle AT/RT indicates a potentially more favorable outcome compared to the general AT/RT population. The relatively limited involvement of lateral ventricle lesions may make maximal resection more feasible, thereby serving as a prognostic modulator and enhancing overall survival potential. This finding is strongly supported by evidence, with GTR as a key factor in improving overall survival in AT/RT. Postoperative adjuvant strategies are varied and typically include high-dose induction chemotherapy, intrathecal and intraventricular chemotherapy, localized or craniospinal irradiation, and stem cell transplantation. This combined multimodal approach has consistently demonstrated superior efficacy compared with monotherapy across multiple clinical cohorts, underscoring its pivotal role in improving the overall prognosis of AT/RT [29,30,31,32]. All 14 cases reviewed in this study were confirmed as primary lateral ventricle AT/RT, excluding metastatic lesions. While an 8-month follow-up is relatively short for assessing definitive long-term outcomes in AT/RT, the limited sample size and the loss of follow-up data for three historical cases further impede accurate survival estimation and introduce potential selection bias. Thus, this hypothesized locational advantage necessitates validation with larger, more comprehensive datasets.

AT/RT are typically resistant to standard chemotherapy and radiotherapy. Radiation therapy can lead to lasting neurocognitive effects in children under three years of age, significantly affecting their quality of life [32,33]. Owing to the aggressive recurrence pattern of AT/RT, novel therapeutic strategies are critically warranted. Identification of AT/RT subtypes, along with their genetic and immune expression characteristics, may facilitate subtype-specific immunotherapy and targeted therapy [16,17,32,33,34,35]. While these therapies hold the potential to modulate the immune environment and suggest a novel avenue for salvage treatment, their overall efficacy in AT/RT remains inconsistent. Current evidence, including preliminary clinical reports, indicates that while some patients may achieve disease stabilization, the challenge of achieving sustained long-term remission through acquired immunity persists [36]. However, several limitations must be considered when interpreting our findings. Given the limited sample size (*n* = 14) and high heterogeneity in adjuvant treatments (including ChT, RT, GKS, and SCT), no definitive prognostic factors could be identified.

The precise pathogenesis of AT/RT is not yet fully understood. Genomic studies have uncovered a relatively simple cancer genome characterized by biallelic mutations, including partial or complete deletions of chromosome 22, leading to the inactivation of *SMARCB1* (a subunit of the SWI/SNF complex). This inactivation represents the primary, and often the sole recurrent molecular feature in AT/RT [15,16,30,37,38,39]. A small proportion of AT/RT cases exhibit mutations in *SMARCA4* [40]. Research on AT/RT extends beyond epigenetics. Johann et al. analyzed 192 histologically confirmed AT/RT samples to investigate DNA methylation and gene expression profiles associated with *SMARCB1* loss [15]. The study identified at least three molecularly distinct and clinically relevant subtypes of *SMARCB1*-deficient AT/RT: AT/RT-*TYR*, AT/RT-*SHH*, and AT/RT-*MYC*, along with a potentially distinct subtype of *SMARCA4*-deficient AT/RT. Each subtype originates from a distinct cell type with a different prognosis [17,23,32].

Current research indicates that the *TYR* subgroup has the most favorable prognosis, while the *MYC* subgroup has the poorest clinical outcomes [17,23,41]. AT/RT-*MYC* is characterized by widespread *SMARCB1* mutations and *MYC* oncogene overexpression, typically occurring in older children (>27 months), with tumors frequently in the supratentorial region, such as the cerebral cortex [17,37,38]. MRI shows significant peritumoral edema wider than other subtypes, possibly due to more growth space in the lateral ventricle, thereby facilitating faster local tumor progression [23]. In the present case, MRI also revealed significant peritumoral edema, consistent with previous reports. Although methylation profiling was not performed for Case 1, the observed homozygous deletion of *SMARCB1* (CN = 0.44) via NGS, coupled with the characteristic radiological features, highly speculates that this case belongs to the AT/RT-*MYC* subgroup. Notably, while AT/RT-*MYC* is generally associated with the supratentorial region, its primary occurrence in the lateral ventricle is exceedingly rare.

The lateral ventricle contains the SVZ, a critical neurogenic niche of neural stem cells (NSCs) and progenitor cells [42]. AT/RT molecular subtypes are hypothesized to originate from cell lineages at different developmental stages. Consequently, investigating the correlation between anatomical location and molecular origin of the tumor has emerged as a frontier research area in the AT/RT field. Lobón-Iglesias et al. successfully defined distinct neural progenitor origins for AT/RT-*SHH* subgroups by integrating imaging and multi-omics datasets. This work identified a subgroup (BG/IV *SHH* AT/RT) associated with tumors in the basal ganglia or intraventricular locations and characterized by differential expression of transcription factors [17]. This finding strongly supports the premise that the anatomical site of AT/RT is not a stochastic event, but rather is intimately linked to the tumor’s molecular features and cellular lineage of origin. It is hypothesized that the unique microenvironment of the SVZ may harbor a specific subtype of precursor cells. They are highly susceptible to *MYC* pathway upregulation upon *SMARCB1* inactivation, thereby driving the tumor toward the highly aggressive *MYC* molecular subtype. This rare presentation of AT/RT primary to the lateral ventricle suggests that a spatially defined, *MYC*-driven progenitor cell exists within this specific neurogenic zone. The favorable prognosis observed in lateral ventricular AT/RT may not stem solely from the ‘locational advantage’ that facilitates gross total resection (GTR). A more profound determinant likely lies in its distinct biological programming. As emphasized in the 5th WHO Classification of Central Nervous System, specific molecular and genetic patterns fundamentally dictate tumor behavior [2]. Thus, the *MYC*-subtype originating from the SVZ may possess unique intrinsic characteristics. Consequently, the survival benefit seen clinically is likely a synergistic effect—a convergence of the endogenous biological features of SVZ-derived cells and the enhanced surgical accessibility afforded by the intraventricular location. This critical hypothesis warrants further validation through advanced techniques such as single-cell sequencing and developmental biology models.

Different molecular subtypes exhibit significant variations in molecular characteristics and immune biology [36]. AT/RT-*MYC* tumors exhibit a higher tumor-infiltrating lymphocytes count, particularly CD8^+^ T cells, compared to other subtypes. Additionally, the upregulation of genes associated with tumor cytokines and IFN-γ expression in AT/RT-*MYC* suggests potential immunotherapy sensitivity [38,43]. However, these CD8^+^ T cells exhibit an exhausted state, potentially affecting their antitumor efficacy, indicating an immunosuppressive tumor microenvironment [34]. Prior research indicates that B7-H3 is universally expressed in all AT/RT subtypes, with high expression inhibiting the tumor immune response and promoting metastasis [44]. B7-H3 targeted CAR-T cell therapy for AT/RT has shown promise in preclinical trials. Theruvath et al. established an AT/RT-*MYC* mouse model and observed that CAR-T therapy reduced tumor size. Furthermore, no tumor progression was noted in the CAR-T treatment group 40 days post-cure upon reimplantation of tumor cells [44]. Vejmelkova et al. reported cases of two pediatric AT/RT patients experiencing tumor recurrence and progression post-conventional surgery with chemoradiotherapy [45]. Subsequent treatment with immunotherapy and Tazemetostat did not prevent tumor metastasis or recurrence, resulting in event-free survival of 5 and 8 months and OS of 20 and 37 months. Despite succumbing to tumor progression, the patients’ OS significantly exceeded the median survival for posterior fossa AT/RT, suggesting a potential adjuvant role for immunotherapy in AT/RT treatment.

In previous studies on AT/RT-*MYC*, PD-1 and PD-L1 were expressed at moderate or higher levels. PD-1/PD-L1 inhibitors primarily function by blocking the interaction between the immune checkpoint protein PD-1, expressed on T cells, and its ligand PD-L1 [36,37]. This inhibition restores T cell functionality and enhances antitumor immune responses. Pembrolizumab, an FDA-approved PD-1 inhibitor for metastatic melanoma and non-small cell lung cancer in combination with chemotherapy, has demonstrated improved progression-free survival in some solid tumors, including brain tumors [46]. Tumor mutational burden (TMB), defined as the total number of somatic mutations per megabase of genomic sequence, and microsatellite instability (MSI), which reflects a high rate of mutations in short, repeated DNA sequences due to a deficient mismatch repair system, are well-established predictive biomarkers for immune checkpoint inhibitor (ICI) efficacy. Despite low TMB and stable MSI, typical features of AT/RT, the high CD8^+^ T cell infiltration in the AT/RT-*MYC* subtype suggests potential immunotherapy sensitivity, ongoing trials like NCT05286801 suggest that chromatin remodeling defects from *SMARCB1* loss may create a unique immune vulnerability. This indicates that traditional TMB markers may not be the sole predictor of efficacy; instead, molecular subtypes (e.g., *MYC*-type) and their specific tumor microenvironments likely play a more critical role.” The high CD8^+^ T-cell infiltration observed in the AT/RT-*MYC* subtype suggests a potential ‘hot’ tumor phenotype. However, the concurrent exhausted state of these lymphocytes indicates a complex immunosuppressive microenvironment that may not respond to PD-1 blockade alone. The integration of ipilimumab (anti-CTLA-4) was intended to enhance T-cell priming and expansion, providing a stronger clinical rationale for the dual-ICI regimen, especially in the context of the low TMB and stable MSI typically found in AT/RT. In our treatment protocol, bevacizumab was included not only for its anti-edema effects but also for its immunomodulatory potential. VEGF signaling contributes significantly to immune evasion by impairing T-cell trafficking to the tumor. Therefore, we hypothesized that VEGF inhibition could synergistically enhance the efficacy of CTLA-4/PD-1 blockade by remodeling the tumor vasculature and promoting a more favorable immune-infiltrative environment. Following chemotherapy with four cycles of pembrolizumab and two cycles of bevacizumab, the patient received ipilimumab. Ipilimumab inhibits CTLA-4 (cytotoxic T-lymphocyte antigen 4), thereby promoting T-cell activation and proliferation to enhance antitumor immune responses [47,48]. Simultaneous blockade of CTLA-4 and PD-L1 can enhance therapeutic efficacy by overcoming resistance to single immune checkpoint inhibition [48,49]. The potential of combined immune checkpoint inhibitors (ICIs) in pediatric INI1-deficient tumors has gained international attention. For instance, the NCT04416568 trial is currently evaluating the safety of combined nivolumab and ipilimumab in such rare tumors, providing a strong clinical rationale for the dual-ICI regimen used in our case. During an 8-month follow-up, the patient experienced occasional headaches, but MRI scans did not reveal disease progression in spinal disseminated lesions. Previous studies by Sharma et al. and Gopalakrishnan et al. reported that pediatric patients with spinal leptomeningeal dissemination during craniotomy had a survival of less than 4 months, suggesting the need for a potentially effective treatment for AT/RT-*MYC* [10,12]. Although Case 1 showed favorable short-term stability, we must consider the multimodal nature of the treatment. Crucially, gross total resection (GTR) combined with local radiotherapy played the fundamental role in securing local control of the primary tumor bed. Radiotherapy may have primed the immune system, while bevacizumab likely controlled peri-tumoral edema and stabilized the blood–brain barrier. The observed clinical stability represents the synergistic outcome of combined salvage interventions, and the specific contribution of each component—particularly immune checkpoint inhibitors versus bevacizumab or prior radiotherapy—cannot be definitively isolated.

In recent years, molecular targeted therapy has been explored as a potentially effective treatment for AT/RT by identifying molecular characteristics and intervening in specific signaling pathways or gene mutations [50]. Studies indicate that *EZH2* mutations or increased activity have an oncogenic role in various tumors, including lymphoma and AT/RT, by promoting cell proliferation [37,38]. In AT/RT-*MYC*, loss of *SMARCB1* leads to increased activity of PRC2 (Polycomb Repressive Complex 2), which includes the catalytic subunit *EZH2*, resulting in the trimethylation and acetylation of histone H3 [51,52]. Preclinical studies have shown that small-molecule *EZH2* inhibitors (e.g., tazemetostat) or genetic knockout can impede cell growth in hematologic malignancies and some solid tumors [52]. Yukitomo et al. reported that the *EZH2* inhibitor GSK126, in combination with the *BRD4* inhibitor JQ1, reduces levels of H3K27me3 and H3K27ac in AT/RT, suppressing tumor cell proliferation and invasiveness, while also mediating resistance to *EZH2* inhibitors [51]. These findings suggest a potential therapeutic strategy of dual targeting *EZH2* and *BRD4* for AT/RT patients, particularly those with refractory or relapsed disease.

In the present study, mutations in the *NF2* and *LZTR1* genes were identified, which is a notable finding. While the *LZTR1* gene has been primarily associated with schwannomatosis, and a direct causal link between *LZTR1* and AT/RT has not yet been established in the literature, current evidence identifies it as a critical tumor suppressor gene. Its germline mutations may pre-dispose individuals to tumor predisposition syndromes, potentially through the dysregulation of the RAS signaling pathway [53]. In our case, the identification of a germline *LZTR1* variant, consistent with the patient’s significant family history, underscores its potential role in the genetic landscape of AT/RT [53,54,55]. At present, no research provides a direct link between the *LZTR1* gene and the development of AT/RT. The *NF2* gene was first identified in adult AT/RT cases in 2005 by Raisanen et al. [56]. Current evidence suggests that this gene is strongly associated with the pathogenesis of schwannomas [54]. However, the potential pathogenic role of *NF2* in AT/RT, particularly at rare sites, remains to be investigated. Lapatinib has shown inhibitory effects on tumors with *NF2* mutations [54]. Its use as a neoadjuvant therapy prior to radiotherapy in young children presents a possible therapeutic avenue to improve survival outcomes.

## 5. Conclusions

AT/RT from the lateral ventricle are exceptionally uncommon, with only 14 cases documented in the literature. Recent research has revealed the epigenetic variations in AT/RT, laying the groundwork for personalized treatment approaches guided by gene expression profiles of distinct molecular subtypes. Combining gene expression analysis and immune surveillance, as illustrated in our case series, underscores the potential of targeted treatments and immunotherapies to enhance outcomes for patients with lateral ventricle AT/RT. The continual investigation of molecularly tailored therapies holds great promise for improving the prognosis of individuals affected by this rare and severe condition.

## 6. Limitation

Despite the clinical insights gained from this study, several limitations must be acknowledged. First, the retrospective nature and the small sample size of 14 cases—comprising three original cases and 11 historical cases from diverse literature sources—limit the statistical power to perform formal correlation analyses or draw definitive prognostic conclusions. The inherent heterogeneity of data reported in the literature may also introduce selection bias. Second, there is a lack of comprehensive molecular data for the two historical cases (case 2 and 3) from our center, as advanced sequencing was constrained by the diagnostic standards of the time and family socio-economic factors. Furthermore, for Case 1, although genomic sequencing (NGS) was performed, specific immune-related biomarkers—such as PD-L1 expression, CD8^+^ tumor-infiltrating lymphocytes (TILs), and B7-H3 levels. Third, regarding the immunotherapy exploration in Case 1, the concomitant use of bevacizumab alongside dual-ICI therapy introduces a potential confounding variable, making it challenging to isolate the precise therapeutic contribution of each agent. Fourth, due to the retrospective nature of this study, telephone follow-ups were used to confirm overall survival, whereas tumor remission was objectively assessed via outpatient MRI. This retrospective approach limits our ability to capture the exact timing of sub-clinical progression. Finally, although Case 1 achieved an 8-month PFS, this follow-up period is relatively short; ongoing monitoring is essential to evaluate the long-term efficacy and safety of this novel regimen in pediatric patients with lateral ventricular AT/RT.

## Figures and Tables

**Table 1:** Summary of 14 cases of pediatric lateral ventricle Atypical Teratoid/Rhabdoid Tumor.

Authors and Year	Age	Sex	Symptoms	Metastases	Extent of Resection	Other Treatment	Recurrence	Outcome	Follow-Up
Donovan et al., 2006 [3]	3M	F	Vomit, seizure	No	STR	ChT	No	Alive	48M
Meyers et al., 2006 [4]	4Y	F	NM	No	STR	ChT + RT	NM	Dead	NM
Lee et al., 2009 [5]	1.3Y	M	Asymptomatic	No	GTR	No	NM	NM	NM
Li et al., 2012 [6]	3Y	M	Headache, vomit, weakness	NM	GTR	RT	NM	Dead	2Y
Darmoul et al., 2015 [7]	2M	M	Macrocephaly	No	GTR	ChT	No	Alive	42M
Singh et al., 2016 [8]	4Y	F	Headache, vomit, seizure	NM	GTR	ChT + RT	NM	NM	NM
Lakhdar et al., 2022 [9]	4Y	M	Hypertension	No	GTR	ChT + RT + GKS	Yes	Dead	23M
Sharma et al., 2020 [10]	4Y	M	Headache, vomit, seizure	Yes	GTR	No	Yes	Dead	3M
Karim et al., 2022 [11]	15Y	M	Headache, nausea, vomit	No	STR	ChT + RT	Yes	Alive	21M
Viswanathan et al., 2023 [12]	3Y	F	Headache, vomit	Yes	GTR	No	NM	Dead	2M
Guo et al., 2022 [13]	19M	M	Claudication	NM	GTR	ChT	Yes	Dead	15M
Case 1	6M	F	Vomit, irritability, seizure	Yes	GTR	ChT + RT + Immunotherapy	No	Alive	8M
Case 2	2Y	M	Headache, vomit	No	GTR	ChT + RT	No	Dead	3M
Case 3	11M	M	Vomit	Yes	GTR	ChT + SCT	No	Alive	36M

Note: Definitions of abbreviations used in the table: M, male; F, female; NM, not mentioned; GTR, gross total resection; STR, subtotal resection; ChT, chemotherapy; RT, radiotherapy; GKS, Radiosurgery gamma knife; SCT, stem cell transplantation.

## Data Availability

The datasets generated and/or analyzed during the current study are available from the corresponding author on request. The data are not publicly available due to patient privacy concerns and institutional policy regarding clinical genetic data.

## Abbreviations

The following abbreviations are used in this manuscript:

AT/RT	Atypical teratoid/rhabdoid tumor
CNS	Central Nervous System
SCNVs	Somatic Copy Number Variations
NGS	Next-Generation Sequencing
OS	Overall Survival
PICU	Pediatric Intensive Care Unit
EVD	External Ventricular Drainage
GTR	Gross Total Resection
STR	Subtotal Resection
DWI	Diffusion-Weighted Imaging
ADC	Apparent Diffusion Coefficient
CPTs	Choroid Plexus Tumors
SVZ	Subventricular Zone
NSCs	Neural Stem Cells
T1WI	T1-Weighted Imaging
ICE	Ifosfamide, Carboplatin, Etoposide
M	Male
F	Female
NM	Not Mentioned
CSI	Craniospinal Irradiation
ChT	Chemotherapy
RT	Radiotherapy
GKS	Radiosurgery Gamma Knife
SCT	Stem Cell Transplantation
TMB	Tumor mutational burden
MSI	Microsatellite Stability
CN	Copy Number
TILs	Tumor-Infiltrating Lymphocytes
